# The anti-tumor diterpene oridonin is a direct inhibitor of Nucleolin in cancer cells

**DOI:** 10.1038/s41598-018-35088-x

**Published:** 2018-11-13

**Authors:** Michele Vasaturo, Roberta Cotugno, Lorenzo Fiengo, Claudio Vinegoni, Fabrizio Dal Piaz, Nunziatina De Tommasi

**Affiliations:** 1 Università degli Studi di Salerno, Department of Pharmacy, Via Giovanni Paolo II, 84084 Fisciano, (SA) Italy; 2 Università degli Studi di Salerno, Ph. D. School of Pharmacy, Via Giovanni Paolo II, 84084 Fisciano, (SA) Italy; 3 Harvard Medical School, MGH Center for Systems Biology, 185 Cambridge Steet, 02144 Boston, MA USA; 4 Università degli Studi di Salerno, Department of Medicine and Surgery, Via S. Allende, 84081 Baronissi, (SA) Italy

## Abstract

The bioactive plant diterpene oridonin displays important pharmacological activities and is widely used in traditional Chinese medicine; however, its molecular mechanism of action is still incompletely described. *In vitro* and *in vivo* data have demonstrated anti-tumor activity of oridonin and its ability to interfere with several cell pathways; however, presently only the molecular chaperone HSP70 has been identified as a direct potential target of this compound. Here, using a combination of different proteomic approaches, innovative Cellular Thermal Shift Assay (CETSA) experiments, and classical biochemical methods, we demonstrate that oridonin interacts with Nucleolin, effectively modulating the activity of this multifunctional protein. The ability of oridonin to target Nucleolin and/or HSP70 could account for the bioactivity profile of this plant diterpene. Recently, Nucleolin has attracted attention as a druggable target, as its diverse functions are implicated in pathological processes such as cancer, inflammation, and viral infection. However, up to now, no small molecule as Nucleolin binders has been reported, thus our finding represents the first evidence of Nucleolin modulation by a small inhibitor.

## Introduction

**Figure 1:**
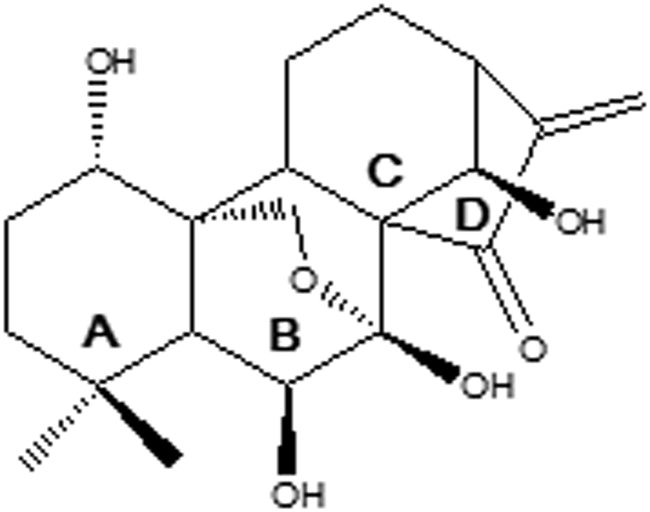
Structure of oridonin; rings A–D were indicated.

Oridonin (Fig. 1), a diterpene extracted from the plant *Rabdosia rubescens* (Hemsl.) Hara (*Donglingcao*) and included in the Chinese Pharmacopoeia in 1977, is used in traditional Chinese medicine as an antitumor, antimicrobial, anti-inflammatory, and antioxidant compound^1^. In the last few decades, oridonin has attracted attention due to its pharmacological properties, as many reports showed that oridonin exerts a broad spectrum of anti-tumoral activities against a number of human cancers and human cancer cell lines such as breast cancer, leukemia, human cervical carcinoma, hepatocellular carcinoma and other tumors^2^. It has also demonstrated a versatile antiproliferative mechanism including regulation of the cell cycle, apoptosis, and autophagy^3^.

Structurally, oridonin is a highly oxygenated 7,20-epoxy-entkaurane-type diterpenoid with an exo-methylenecyclopentanone moiety in the D-ring, in addition to the α,β-unsaturated function, highly functionalized in rings A and B. It has been reported that the α,β -unsaturated ketone function is crucial for anticancer activity of oridonin^4^. On the other hand, many reports have shown that the modification on the A ring is feasible and could represent a way to increase oridonin potency^4^. Based on this evidence, we have hypothesized that the high functionalization of oridonin skeleton could also have a prominent role in its biological activity.

In our previous study^5^, oridonin was identified as a novel inhibitor for the molecular chaperone HSP70 in leukemia-derived Jurkat. Even if HSP70 inhibition affects several pathways, playing a critical role in the stabilization of proteins implicated in the control of cell growth and malignant transformation^6^, the efficiency of oridonin in promoting cell cycle inhibition, apoptosis and autophagy interacting with multiple targets could not be completely explained just by oridonin/HSP70 interaction. Therefore, inspired by oridonin structural features, we embarked on the study of additional oridonin targets and on the investigation of the role of non-covalent interactions in target selection. Two different mass spectrometry-based proteomic approaches were used; affinity chromatography using the immobilized small molecule^7^, which allows to detect the specific interactors in cell lysates, was carried out in combination with the drug affinity responsive target stability (DARTS) technique, whose major advantage is the use of unmodified compounds for target identification^8,9^. Both these approaches confirmed HSP70 as an oridonin target, but also led to the identification of Nucleolin as a cellular interactor of the diterpene. Subsequently, classical and innovative *cell-free* and *cell-based* assays were used to demonstrate oridonin to be an effective Nucleolin modulator in two human cancer-derived cell lines, Jurkat (leukemia T cell line) and HeLa (cervical cancer cell line).

Recently, Nucleolin has attracted attention as a druggable target, as its diverse functions are implicated in pathological processes such as cancer, inflammation, and viral infection^10^. Therefore, Nucleolin inhibitors might represent an emerging therapeutic strategy, but until now no small molecules as Nucleolin binders have been identified. Our data represent the first report of Nucleolin inhibition by a small molecule;, thus throwing the bases for oridonin as the starting point for the development of new drugs as well as a probe to study in depth Nucleolin structure and functions.

## Results

### Oridonin uptake into cancer cells

In order to deepen our previous study^5^ on the mechanism of action of oridonin, the efficiency and the kinetics of oridonin uptake into leukemia-derived Jurkat cells were investigated. For this purpose, we synthetized a fluorescent derivative of the diterpene, using as a fluorescent label BODIPY FL (Supplementary Fig. S1a), a suitable tag for intracellular imaging assays^11^. The obtained fluorescent oridonin (FlOr) demonstrated to retain the same activities of the parent compound. FlOr displayed in Jurkat cells an IC_50_ of 1.45 ± 0.22 μM and 1.15 ± 0.30 μM at 24 h or 48 h treatment, respectively, values substantially comparable to those measured for oridonin (IC_50_ values 1.19 ± 0.13 μM and 0.73 ± 0.20 μM at 24 h or a 48 h treatment) (Supplementary Fig. S1b). Moreover, FlOr maintained the ability to covalently bind HSP70 (Supplementary Fig. S1c).

**Figure 2:**
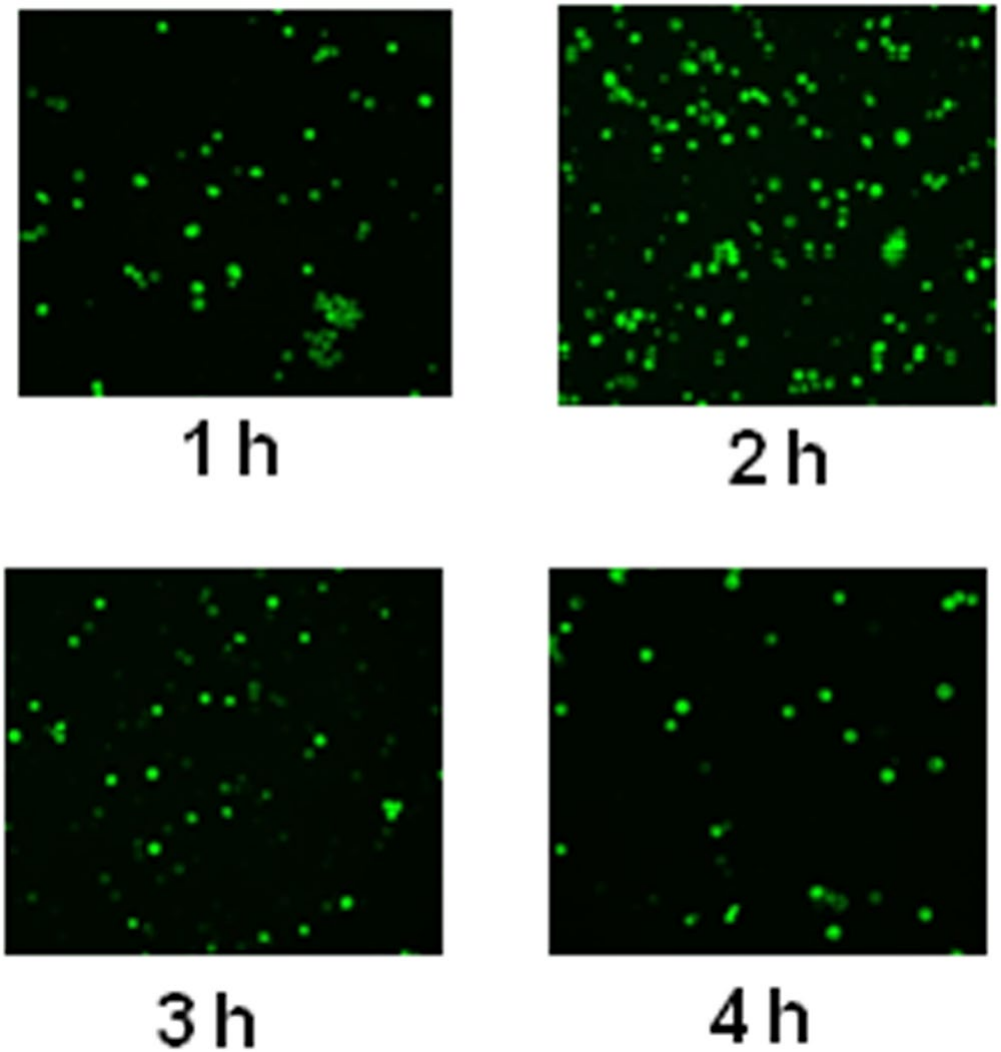
Oridonin uptake kinetics. Jurkat cells were incubated with 5 μM FlOr and the amount of compound inside the cells following different incubation times was evaluated by fluorescence microscopy. Light pictures of the same cells are reported in Supplementary Fig. S2.

To study oridonin uptake, Jurkat cells were incubated with a 5 μM FlOr for different times. Real-time *in vitro* fluorescence microscopy measurements (Fig. 2 and Supplementary Fig. S2) showed that the amount of FlOr into the cells reached its maximum after 2 h; longer exposure times led to lower levels of intracellular FlOr.

### Oridonin target(s) identification

The identification of further putative targets of oridonin was attempted performing DARTS (Drug Affinity Responsive Target Stability) experiments^8,9,12^. This indirect compound-centered proteomic approach is based on the evidence that the effective interaction of a protein with a ligand sensibly reduces the protein susceptibility to proteolysis; DARTS can be considered complementary and alternative to the classic chemical proteomics affinity-based method previously used to investigate oridonin targets^3^, since it allows studying the interactome of a bioactive compound without requiring its chemical modification and/or immobilization.

**Figure 3:**
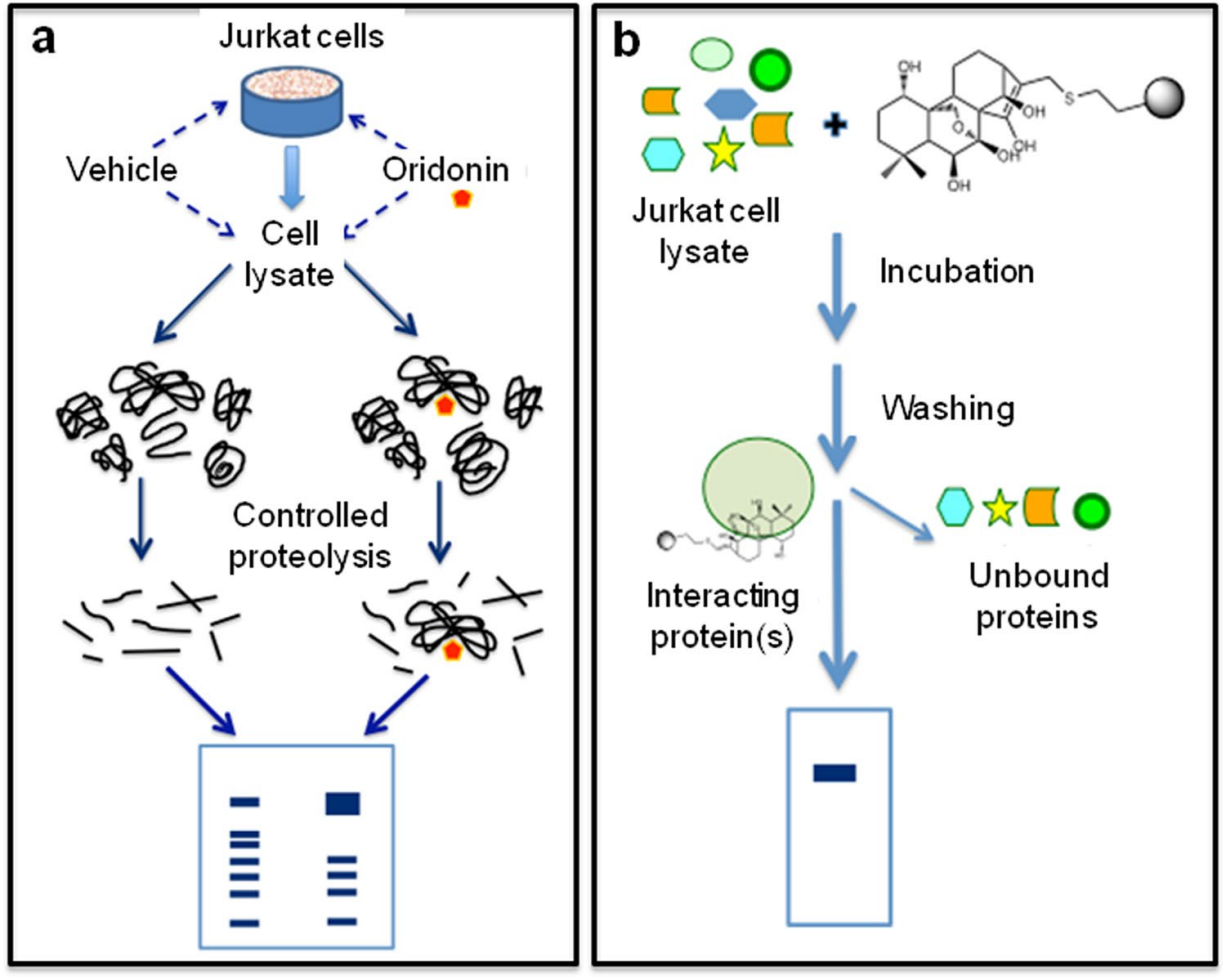
Compound-centric proteomic approaches to attempt the identification of oridonin targets. (**a**) DARTS experiments were performed incubating 5 μM oridonin with Jurkat cell lysates or living Jurkat cells. Protein(s) interacting with oridonin could be identified on the basis of an increased resistance against proteolysis. (**b**) Chemical-proteomics experiments were carried out using immobilized oridonin to perform an affinity-based purification of the protein partner(s) of the diterpene.

**Figure 4:**
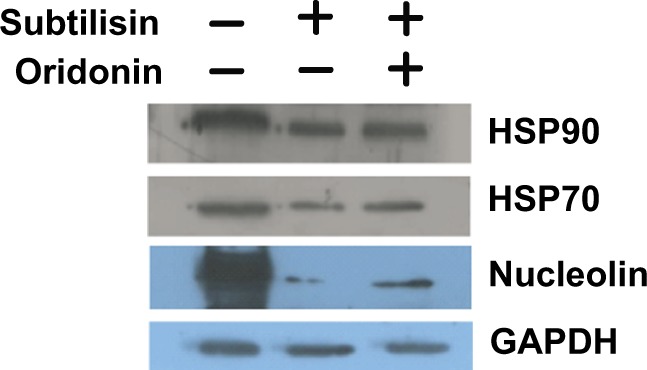
Western blot analysis of DARTS experiments. Protein lysates obtained from Jurkat cells treated with 5 μM oridonin or from untreated cells underwent subtilisin digestion. Resulting protein mixtures were separated on a SDS-PAGE and detected using suitable antibodies. GAPDH was used as a control protein as it resists to subtilisin catalyzed proteolysis. Blots of HSP90, HSP70, Nucleolin and GAPDH were taken from different gels.

We carried out DARTS experiments both on cell lysates and on living cells (Fig. 3a). In the first case, protein extracts, obtained from Jurkat cells under non-denaturing conditions, were incubated with 5 µM oridonin or with DMSO for 1 h and then subjected to a limited digestion with subtilisin. The resulting partially hydrolyzed protein mixtures were separated by SDS-Page. The occurrence in the lanes of oridonin-treated lysate of gel bands showing a higher intensity than the corresponding ones in the control lane (Supplementary Fig. S3a), suggested the presence of proteins protected from proteolysis by the interaction with the diterpene. Those bands were excised from the gel and subjected to a trypsin in-gel digestion procedure, followed by nanoUPLC-hrMS/MS analyses of the resulting peptides mixtures. A bio-informatics analysis of the spectrometric data led to the identification of eleven proteins (Supplementary Table S1), which remained partially undigested in the oridonin-treated sample and largely hydrolyzed in the untreated one. Subsequently, DARTS experiments were performed in Jurkat whole cells, exposed to 5 µM oridonin or DMSO. After 2 h of incubation, proteins were extracted under non-denaturing conditions and underwent subtilisin-catalyzed hydrolysis. The obtained partially digested protein mixtures (Supplementary Fig. S3b) were analyzed using the same protocol discussed before. In this case, seven “protected” proteins were identified (Supplementary Table S2). Comparing the results achieved using the two approaches, only three proteins emerged as putative oridonin targets: HSP70, HSP90, and Nucleolin. This result was confirmed performing a further DARTS experiment on intact cells, and analyzing the partially digested protein mixtures by western blot (Fig. 4).

The identification of HSP70 with DARTS approach further confirmed the efficiency of this strategy; on the other hand, our previous study demonstrated that HSP90 is not an oridonin target^5^, and it probably emerged from the DARTS experiments because of its stable interaction with HSP70^13^. Thus, the most promising result achieved by DARTS experiments was the indication of Nucleolin as a putative oridonin target. However, it remains to be clarified why this protein did not emerge from our previous affinity-based chemical proteomics experiments. Possibly, the chemical modification of oridonin introduced on C-1 to perform those studies significantly affected the affinity of the diterpene towards Nucleolin. To validate this hypothesis, and to confirm the interaction between oridonin and Nucleolin, we performed a new affinity-based chemical proteomics experiment modifying the α,β unsaturated carboxyl-group. This modification prevented covalent binding of oridonin with its putative targets, but it allowed identifying the proteins capable of establishing efficient non-covalent interaction with the polycyclic portion of the molecule. Therefore, oridonin was immobilized on a TentaGel resin and incubated with a Jurkat lysate (Fig. 3b). Cell extract was also incubated with a β-mercaptoethanol-modified resin, as a control experiment, to distinguish between specifically bound components and background contaminants. Identification of tightly bound proteins was performed following a classical chemical proteomics protocol^7^, leading to the definition of a list of proteins interacting with the immobilized oridonin (Table 1), interestingly including Nucleolin and HSP70. The presence in this list of three different heterogeneous nuclear ribonucleoproteins (hnRPN) and of the ribosomal protein 60S represented a further confirmation of the ability of oridonin to bind Nucleolin; indeed, several evidences of direct and indirect interactions between Nucleolin and hnRPNs and between Nucleolin and 60S have been reported^14–17^. Therefore, these proteins were possibly “fished” by the oridonin-modified resin as a consequence of their binding to Nucleolin.

**Table 1:** Putative oridonin-interacting proteins emerged from chemical-proteomics experiments.

Swiss-Prot CODE	Score^a^	Protein	Peptides^b^
NUCL_HUMAN	528	Nucleolin	9
HNRPH1_HUMAN	500	Heterogeneous nuclear ribonucleoprotein H1	5
HNRPF_HUMAN	493	Heterogeneous nuclear ribonucleoprotein F	5
HNRPD_HUMAN	389	Heterogeneous nuclear ribonucleoprotein D_0_	4
HSP71A/HSP71B_HUMAN	287	Heat shock 70 kDa protein 1A/1B	4
RPLP0_HUMAN	160	60S acidic ribosomal protein P0	3

^a^Average score value achieved in three different chemical-proteomics experiments.

^b^Number of unique peptide sequences detected in a single experiment. Reported values are the average of the unique sequences detected in three different chemical-proteomics experiments.

Nucleolin is a ubiquitously expressed acidic phosphoprotein with key functions in transcription, mRNA stabilization, synthesis and maturation of ribosomes, and it is involved in critical aspects of cell growth and proliferation^18^. It is localized primarily to the nucleoli, but it can be found in nucleoplasm and cytoplasm^19^. In highly proliferating cells it also moves onto the plasma membrane and this translocation, regulated by HSP70, has been reported to have a pivotal role in the induction of angiogenesis in cancer cells^20^. Nucleolin plays a role in stabilizing the mRNAs of Akt, Bcl-2, Bcl-xl, and IL-2, and it is also involved in regulating multiple apoptosis-related molecules. Moreover, Nucleolin levels and intracellular localization are frequently altered in cancer and cancer-associated endothelial cells^15^. The multiple biological activities of Nucleolin in cancer cells, and the functional relationship involving Nucleolin and HSP70 in cancer-related angiogenesis, suggested that oridonin effects on cancer cells could depend on the simultaneous inhibition of these two proteins. Thus we performed a multiple approach study aimed to validate Nucleolin as oridonin target and to evaluate the effects of oridonin treatments on Nucleolin activity.

### Oridonin efficiently interacts with Nucleolin

**Figure 5:**
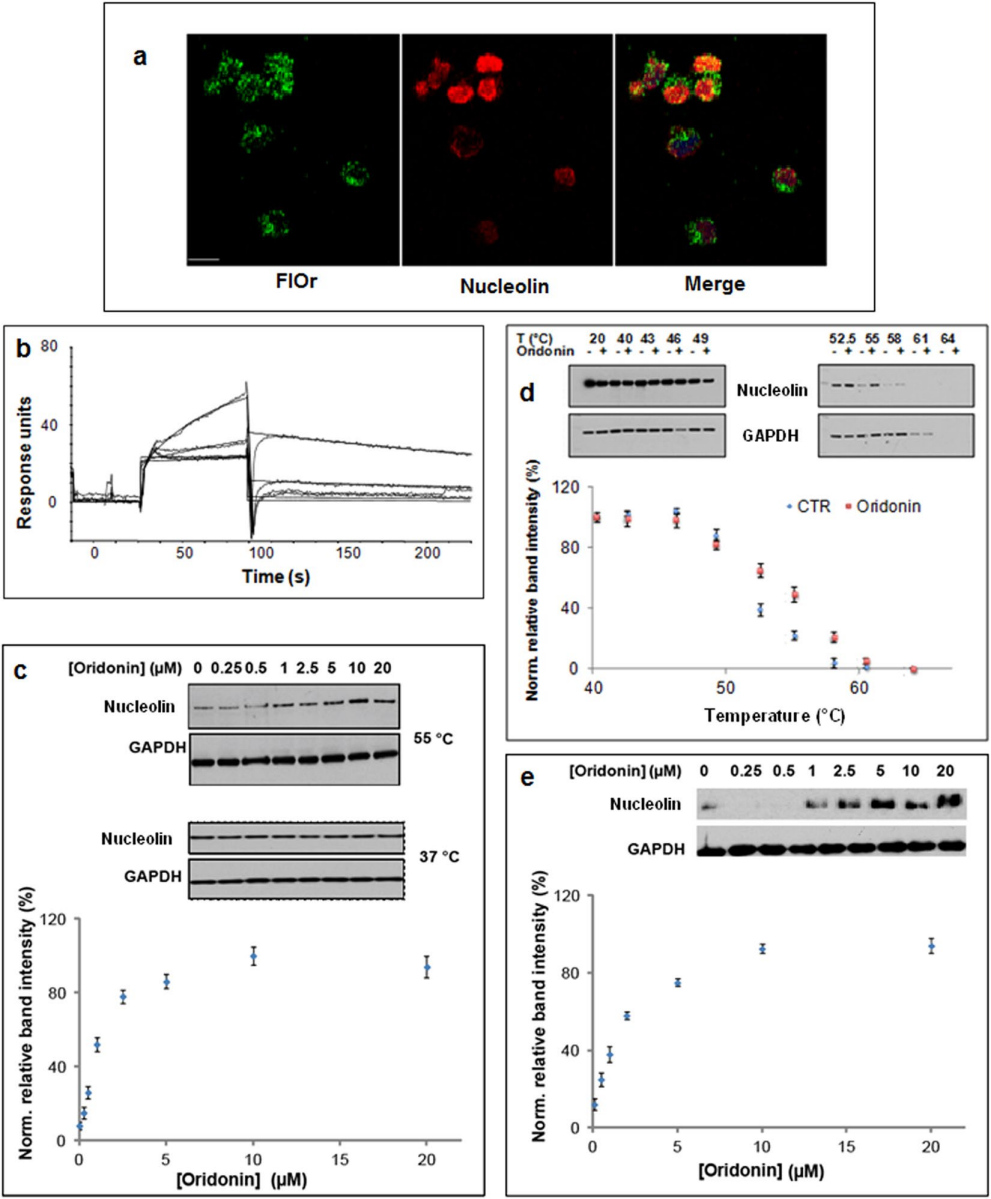
Oridonin efficiently interacts with Nucleolin both *in vitro* and inside the cell. (**a**) FlOr distribution inside Jurkat cells is partially superimposable with that of Nucleolin (**b**) The ability of oridonin to interact with Nucleolin was monitored using surface plasmon resonance: different concentrations (1, 5, 25 and 125 nM) of the diterpene were injected on immobilized Nucleolin. (**c**) CETSA experiments were performed to confirm that this interaction occurred also inside the cells. The effectiveness of oridonin in prevent thermal denaturation of Nucleolin was monitored treating Jurkat cells with 5 µM oridonin and then incubating these cells at different temperatures. (Upper) Representative WB analysis of Nucleolin levels in control (−) and in oridonin-treated Jurkat cells. GAPDH was used as loading control. (Lower) Densitometry data from 3 separate experiments are plotted. Values are reported as mean ± SD. (**d**) Oridonin concentration providing half of the maximum stabilizing effect on Nucleolin (EC_50_) was defined by treating Jurkat cells with variable amounts of the diterpene and performing CETSA protocol. (Upper) Representative WB analysis of Nucleolin levels in control (−) and in oridonin-treated Jurkat cells. GAPDH was used as loading control. (Lower) Densitometry data from 3 separate experiments are plotted. Values are reported as mean ± SD. Possible effects on the stabilization of Nucleolin by oridonin were also evaluated at 37 °C. (**e**) Oridonin concentration providing half of the maximum stabilizing effect on Nucleolin (EC_50_) was defined by treating HeLa cells with variable amounts of the diterpene and performing CETSA protocol. (Upper) Representative WB analysis of Nucleolin levels in control (−) and in oridonin-treated Jurkat cells. GAPDH was used as loading control. (Lower) Densitometry data from 3 separate experiments are plotted. Values are reported as mean ± SD. Blots of Nucleolin and GAPDH were taken from different gels.

The hypothesis of a direct interaction between oridonin and Nucleolin in Jurkat cells was supported by a co-localization experiment carried out using FlOr (Fig. 5a). Subsequently, the affinity of unmodified oridonin to Nucleolin was evaluated by a Surface Plasmon Resonance (SPR)-based binding assay^21^. Oridonin was injected on a Nucleolin-modified sensor chip at concentrations ranging from 1 to 625 nM; the resulting sensorgrams (Fig. 5b) clearly confirmed the effective interaction between oridonin and immobilized Nucleolin, and a dissociation constant (K_D_) of 3.8 ± 1.2 nM was measured for the Nucleolin/oridonin complex. Although the observed K_D_ indicated the occurrence of a stable interaction between the protein and the diterpene, a clear dissociation phase can be observed in the sensorgrams, thus suggesting that no covalent bond was formed. To support this hypothesis, recombinant Nucleolin was incubated with oridonin under different experimental conditions and subjected to MS-based peptide mapping analyses; although most of the expected peptides were detected, no modified species was observed (Supplementary Table S3).

To get more detailed information on oridonin and Nucleolin interaction and to study the complex inside the living cells, CETSA (Cellular Thermal Shift Assay) experiments were carried out^22^. The possible shift in thermal stability of Nucleolin following oridonin binding was monitored by measuring the amount of protein remaining soluble at different temperatures inside oridonin-treated and control Jurkat cells. Preliminarily, to define the optimal temperature to detect the potential stabilization effect of the interaction (T_agg_), we treated the cells with 5 µM oridonin or with the vehicle for 2 h, and divided them into 10 aliquots; each aliquot was then exposed to a defined temperature (in the range from 40 to 68 °C). After cooling, a quantitative analysis of soluble Nucleolin was performed by western blotting, using gliceraldeide-3-phosphate dehydrogenase (GAPDH) as a control (Fig. 5c upper). The graph in Fig. 5c (lower), reporting soluble Nucleolin vs incubation temperature, shows the significant thermal stabilization of Nucleolin exposed to oridonin treatment of Jurkat cells. This result clearly supports the formation of an oridonin-Nucleolin complex inside Jurkat cells. In particular, 55 °C could be set as T_agg_, since following the incubation at this temperature the soluble amount of Nucleolin in treated cells was nearly double than that measured in control cells. As expected, T_agg_ almost corresponded to the denaturation temperature of Nucleolin in the untreated Jurkat cells. Subsequently, a time-course CESTA experiment was performed to define the kinetics of Nucleolin stabilization by oridonin treatment. Jurkat cells were incubated for different times with 5 µM oridonin and the previously described CETSA protocol was performed. Soluble Nucleolin vs incubation time resulting graph (Supplementary Fig. S4) suggested the maximum of Nucleolin stabilization would occur following 2 h of incubation. Taking advantage of defined T_agg_ and optimal incubation time, we carried out an Isothermal Dose-Response Fingerprints Cellular Thermal Shift Assay (ITDFCETSA) experiment to establish the half-maximal effective dose (EC_50_) of oridonin. Jurkat cells were treated with different concentrations of oridonin up to 20 µM, while incubation time (2 h) and cell incubation temperature (55 °C) were kept constant (Fig. 5d). A clear dose-dependent response was observed, with the oridonin concentration providing the higher stabilizing effect on Nucleolin at 10 µM. From these data, the oridonin concentration producing half of the maximum effect on protein stabilization (EC_50_) was 0.9 ± 0.2 µM.

To evaluate whether the formation of oridonin-Nucleolin complex was cell line-specific, we performed some experiments also in HeLa cells. We first calculated the oridonin EC_50_ value in HeLa cells (1.6 ± 0.1 μM), which resulted only slightly higher than that measured in Jurkat cells. Then, the ability of oridonin to interact with Nucleolin in HeLa cells was investigated by CETSA experiments (Fig. 5e). As expected, the measured T_agg_ for Nucleolin in these cells (55.5 °C) was not far from that observed in Jurkat cells. Our results demonstrated that oridonin was able to stabilize Nucleolin also inside HeLa cells.

### Effects of oridonin on Nucleolin activities

**Figure 6:**
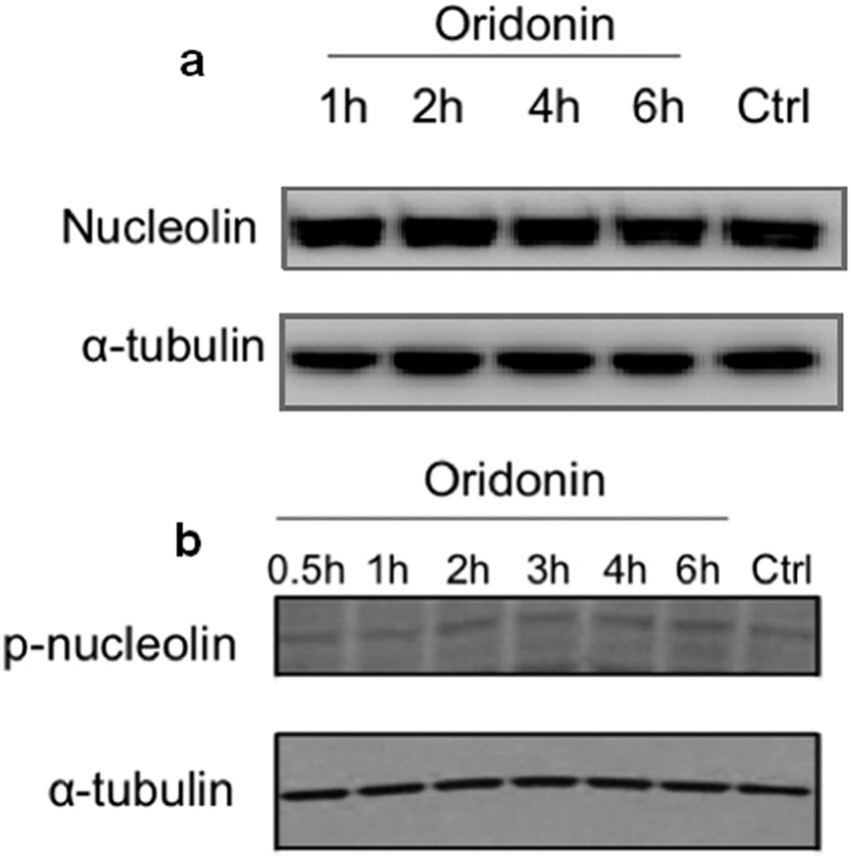
Effects of oridonin on Nucleolin intracellular level and post-translational modification. (**a**) Nucleolin and (**b**) p-Nucleolin levels in Jurkat cells exposed to 5 µM for increasing time. α-tubulin was used as loading control. Blots shown are from one experiment representative of at least three with similar results. Blots of Nucleolin, p-Nucleolin and α-tubulin were taken from different gels.

The possible consequences of oridonin treatment on the Nucleolin levels were monitored, but no significant differences were observed between treated and untreated cells, till 6 h of exposure to 5 µM oridonin (Fig. 6a). Similar results were achieved when the levels of phosphorylated Nucleolin (p-Nucleolin) following cells incubation with 5 µM oridonin were investigated (Fig. 6b), thus indicating that oridonin has no significant effects on both Nucleolin expression and post-translational modification.

**Figure 7:**
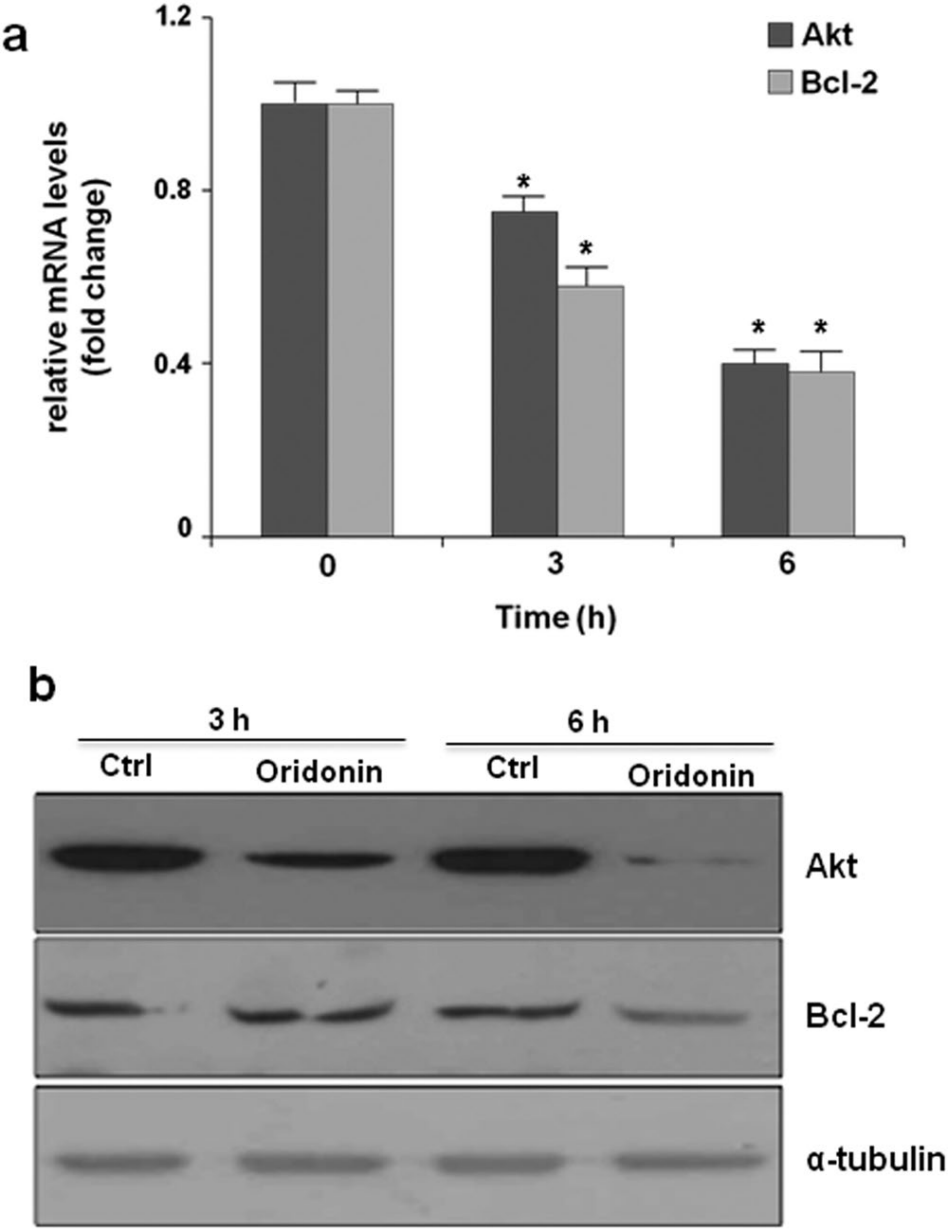
Oridonin effect on Nucleolin mRNA stabilization activity. (**a**) Akt and Bcl-2 mRNA level in Jurkat cells treated with 5 µM oridonin for 3 and 6 h. Data are the mean values ± SD from three independent experiments performed in duplicate (n = 6). *p < 0.05 vs Time 0 (**b**) Akt and Bcl-2 level in Jurkat cells exposed to 5 µM oridonin for 3 and 6 h; α-tubulin was used as loading control. Blots shown are from one experiment representative of three with similar results. Blots of Akt, Bcl2 and α-tubulin were taken from different gels.

We then focused our attention on two of the most crucial roles played by this protein in proliferating cells: its mRNA stabilizer activity^23^, and its involvement in ribosome assembly^24^. Nucleolin modulates the expression of several proteins involved in malignant cells survival through its mRNA binding activity, conferred by four RNA binding domains. Nucleolin affects mRNA turnover by interacting with the 3′-untranslated region of several target mRNAs. In particular, since Nucleolin has been reported to bind the mRNAs encoding for Bcl2^25^ and Akt^26^, its inhibition could result in a destabilization of these two mRNA and, consequently, in a decrement of the amount of the corresponding proteins. Accordingly, by means of qRT-PCR (Real-Time Quantitative Polymerase Chain Reaction) and western blot analysis, we found that Jurkat cells treated with 5 µM oridonin or vehicle for 3 h and 6 h displayed reduced levels of mRNAs of Bcl-2 and Akt (Fig. 7a), as well as reduced levels of the two proteins (Fig. 7b). Although these results suggest that oridonin can impair Nucleolin ability to stabilize specific mRNA, further experiments are required to confirm this hypothesis.

**Figure 8:**
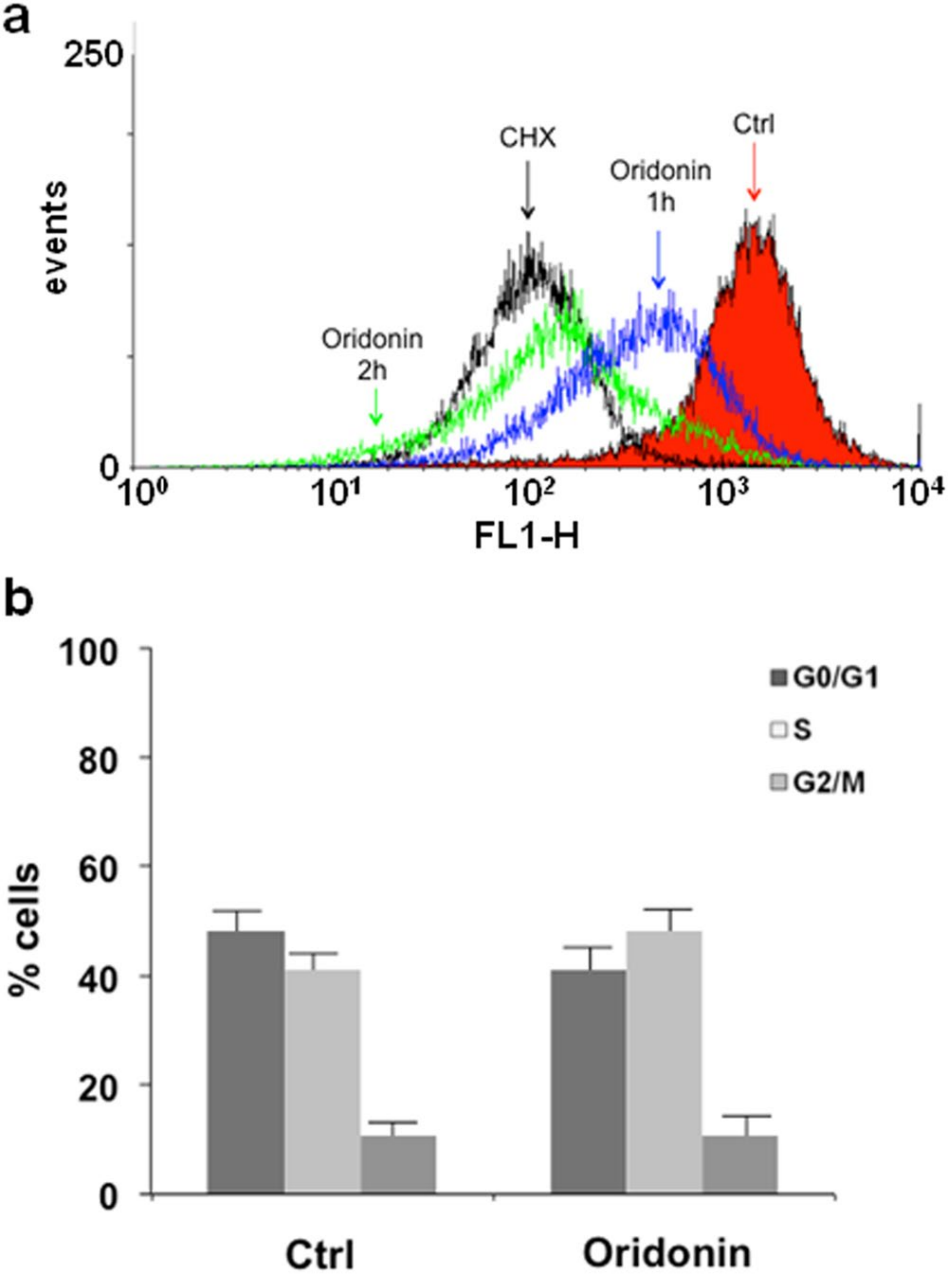
Oridonin effects on protein synthesis and cell cycle progression. (**a**) Jurkat cells were treated with vehicle (red), 50 μg/mL cycloheximide for 30 min (black), or 20 μM oridonin for 1 h (blue) and 2 h (green). Cells were then incubated with OPP. (**b**) Flow cytometric evaluation of DNA content in Jurkat cells exposed for 2 h to oridonin and vehicle alone (Ctrl). Values are the mean ± SD from three experiments.

Nucleolin plays a critical role in several steps of ribosome biosynthesis^24^; this protein is indeed involved in rDNA transcription^27^, is associated with nascent pre-rRNAs^28,29^, helps assembly^30^, and transport^31^ of ribosomal particles. Consequently, Nucleolin impairment by oridonin could have dramatic effects on protein synthesis. We monitored protein neo-synthesis in control cells and in cells undergoing incubation with 5 µM oridonin for 1 and for 2 h. Cycloheximide, a well-known inhibitor of the protein synthesis, was used as the positive control whereas DMSO was the negative one. Following incubation, the samples were treated with Click-iT^®^ OPP (O-Propargyl-Puromycin), a puromycin analog readily taken up by actively growing cells, which inhibits protein synthesis by disrupting peptide transfer on ribosomes and causing premature chain termination during translation. Addition of the 5-fluorescein azide and the click reaction reagents led to a chemoselective ligation between the azide dye and the alkyne OPP, allowing the modified proteins to be detected. Finally, all the samples were analyzed by flow cytometry. As depicted in Fig. 8a, cells treatment with oridonin produced a time-dependent decrement in the observed fluorescence intensity, and after 2 h a signal comparable to that observed for cycloheximide was recorded. This result demonstrated the ability of oridonin to inhibit the protein synthesis process. Since oridonin is able to arrest cell cycle in S-G_2_/M phase^5^, we wondered if the observed inhibition of protein synthesis could depend on this block. Hence, we evaluated Jurkat cells cycle under the experimental conditions adopted to assay protein synthesis; as shown in Fig. 8b the cell cycle profile of oridonin-treated cells was substantially comparable to that of control cells. These experiments were performed also on HeLa cells, providing comparable results (Supplementary Fig. S5).

## Discussion

Nucleolin is a multifunctional protein playing critical roles in mRNA stabilization and protein synthesis^31^, but also in many other cellular processes (i.e. DNA metabolism, repair and duplication^32^, chromatin remodeling^33^, rRNA transcription^34^). It interacts with several protein (HSP70^35^, histone H1^31^, SWI/SNF^31^, hnRNPs^14–16^, and ribosomial proteins^17^), and nucleic-acid partners (DNA; mRNA; pre-rRNA, rDNA)^31^, and is located in different cell regions (nucleolus, cytoplasm and plasma membrane)^36^.

The multiple tasks performed by Nucleolin make this protein crucial for several physiological and pathological processes. In particular, the critical functions of Nucleolin in cancer progression and resistance to therapeutic agents have been largely investigated^19^. This protein stabilizes mRNAs of anti-apoptotic proteins (Bcl-2, Akt, Ccn I)^25,37^, co-operates in DNA repairs^38^, enhances the anti-apoptotic effects of HSP70^39^, and, in combination with HSP70, promotes pathologic angiogenesis^20^. Moreover, there are several evidences indicating that Nucleolin located in plasmatic membrane can facilitate the binding of HIV on host cell surface, and its entrance^40^. Finally, Nucleolin has been shown to promote inflammation, enhancing the internalization of lipopolysaccharides from the cell membrane to the cytosol^41^. Unfortunately, the number of Nucleolin modulators identified is very low, and most of them are selectively addressed towards cell-surface Nucleolin^42,43^. To the best of our knowledge, oridonin is the first identified small molecule inhibiting Nucleolin. This finding opens the way to many possible uses of this diterpene: first, the therapeutic use of oridonin - alone or in combination - to face oncologic pathologies can be optimized. Moreover, since Nucleolin plays a pivotal role in viral infection, oridonin could be assayed also for its possible anti-viral effects. Finally, this compound could also represent an excellent probe to study more in depth such an important protein as Nucleolin.

There is however another interesting result emerging from our studies: the natural compound oridonin can inhibit simultaneously two proteins crucial for cancer development and progression, HSP70 and Nucleolin. This finding could actually explain the efficiency of oridonin as antitumor agent and its ability in interfere with many different pathways^44,45^. The two proteins are functionally related, and their direct interaction has been reported. HSP70 has been shown to prevent degradation and cleavage of Nucleolin in cancer cells following exogenous stresses^35,46^, and to regulate Nucleolin translocation to cell surface^20^. On the other hand, over-expression of Nucleolin is directly related to HSP70 mRNA stabilization^47^, and to an increased anti-apoptotic efficiency of the chaperone^39^. Therefore, the simultaneous inhibition of HSP70 and Nucleolin functions by oridonin could lead to the impairment of several anti-apoptotic and proliferation signal pathways, crucial particularly for cancer cell survival.

Taken together, our results suggest oridonin as a reasonable starting point to obtain small-molecule able to modulate Nucleolin activity. Furthermore, the present discovery of the first small-molecule interactor of Nucleolin provides proof-of-principle evidence for the feasibility of small-molecule modulation of Nucleolin activity, and should lead to new pharmacological tools to study Nucleolin mediated phatologic responses.

## Methods

### Oridonin uptake evaluation

Oridonin (100 mg, 0.27 mmol) was dissolved in acetone (10 ml) and the solution was added of 5 mg tosyl acid and 1 mL of 2,2-dimethoxypropane. The mixture was stirred at room temperature (R.T.) for 15 min, diluted with water and extracted with dichloromethane (DCM). The extract was washed with saturated NaHCO_3_ solution and brine, dried over anhydrous Na_2_SO_4_, filtered, and evaporated. Resulting compound (intermediate 1; 32 mg, 0.0792 mmol) was dissolved in 700 µL of dry DCM and added of dimethylaminopyridine (38.7 mg 0.316 mmol) and 4-nitrophenyl chloroformate (32 mg 0.158 mmol). This mixture was stirred at R.T. for 1 h. After reaction completion, the mixture was dried and the resulting product was purified on silica gel column. The achieved compound (intermediate 2, 10 mg 0.024 mmol) was incubate with BODIPY ammine (26 mg 0.05 mmol) in 900 µL of DCM: dimthylformamide 50:50 (v/v) at R.T. for 1 h. After reaction completion, the mixture was dried and the reaction product (intermediate 3) was purified by HPLC on a C-18 column. In order to get the final product FlOr, intermediate 3 (10 mg 0.014 mmol) was dissolved in 2 mL of tetrahydrofuran 10% HCl and stirred at R.T. for 1 h. HPLC purification lead to the isolation of 6.8 mg of the desired product, whose identity and purity was confirmed by ^1^H and  ^13^C NMR spectra (recorded at 23 °C on Burker 400 MHz spectrometer) and LC-ESI-MS analysis performed on a Waters (Milford, MA, USA) Q-TOF premier LC-MS system (measured *m/z* 681.382, calculated for C_36_H_46_BF_2_N_3_O_7_: 681.341).

The biological activity of FlOr was measured evaluating its anti-proliferative effect on Jurkat cells, and comparing its efficiency with that measured for unmodified oridonin. Jurkat cells were seeded in 96-well microtiter plates in 100 µL of growth medium. After 24 h and 48 h of incubation at 37 °C, cells were exposed to different concentrations of oridonin and FlOr ranging from 0.01 μM to 40 μM or vehicle only (DMSO 0.1%, final concentration). The mitochondrial-dependent reduction of MTT to formazan was used to assess the cytotoxic potential. The experiment was carried out in triplicate and all the values were normalized to control. The ability of FlOr to covalently bind HSP70 was also investigated. Five hundred nanograms of human recombinant HSP70 (GenBank No. NM_005345, Tebu-Bio, Milano, Italy) were incubated with different molar excesses (starting from 0.01 up to 10 molar folds more) of FlOr in PBS at 25 °C under stirring for 15 min. Each sample was loaded on a mono-dimensional 12% SDS-PAGE and stained with Brilliant Blue G-Colloidal. The fluorescence intensity of FlOr into the gel was monitored by Typhoon scanner FLA 7000 (GE Healthcare Life Sciences) using excitation and emission λ of 488 nm and 512 nm, respectively. Jurkat cells were seeded into a 96-well plate at 5 × 103 cells/ml and incubated in growth media containing 5 µM of FlOr and 0.1% DMSO for 1 h up to 4 h. Following the selected incubation time, cells were washed with FBS 10% (v/v), then Hoechst 33342 (ThermoFisher Scientific) was added to each sample for 30 min. Live cells were subsequently imaged on a Delta Vision imaging system (Applied Precision, GE Healthcare). Images were processed using Fiji software, an open-source version of ImageJ.

### DARTS experiments

As a preliminary control, we evaluated if oridonin could interfere with subtilisin intrinsic activity. Therefore, 10 µg recombinant bovine serum albumin (BSA) were incubated with and without oridonin (20 µM, 1% DMSO) in PBS for 1 h at R.T. under stirring, and underwent proteolysis using a 1:1000 (w:w) subtilisin: BSA ratio for 30 min. Reaction mixtures were then resolved by SDS-PAGE gel and stained with Brilliant Blue G-Colloidal; resulting electrophoretic profiles were compared, and no significant difference was observed. Jurkat cells were lysed in RIPA buffer supplemented with protease and phosphatase inhibitors. Protein concentration was determined by DC Protein Assay (Bio-Rad, Berkeley, CA, USA), using BSA as a standard. Lysates (50 μg) were incubated with 2 μL of PBS 8.5% DMSO, or 2 μL of 45 μM oridonin, 8.5% DMSO in PBS, to obtain the final concentration of 5 μM oridonin, 1% DMSO. The samples were incubated for 1 h at R.T. under stirring. After that samples underwent proteolysis with subtilisin (enzyme: lysate 1:1000 w/w) for 30 min; hydrolysis was stopped adding 5 μL of Laemmli buffer 4X and incubating the mixture at 100 °C for 5 min. Samples were loaded on a 10% mono dimensional SDS-PAGE gel. Bands appearing more intense in the treated samples than in the control ones were excised from both the gel lanes and subjected to classical in gel digestion procedure^48^. The resulting fragments were extracted and analyzed by LC/MS/MS using a LTQ Orbitrap XL ESI-mass spectrometer (Thermo Fisher Scientific) equipped with a nano-ESI source, coupled with a nano-Aquity capillary UPLC (Waters): peptides separation was performed on a capillary BEH C18 column (0.075 mm × 100 mm, 1.7 µm, Waters) using aqueous 0.1% formic acid (A) and CH_3_CN containing 0.1% formic acid (B) as mobile phases. Peptides were eluted by means of a linear gradient from 10 to 40% of B over 45 min and a 300 nl/min flow rate. Peptide fragmentation was achieved using helium as collision gas and a collision cell energy of 30 eV. Mass spectra were acquired over a *m/z* range from 400 to 1800, and MS/MS spectra in a *m/z* range from 25–2000. MS and MS/MS data were used by Mascot (Matrix Science) to interrogate the Swiss Prot non-redundant protein database. Settings were as follows: mass accuracy window for parent ion, 10 ppm; mass accuracy window for fragment ions, 50 millimass units; fixed modification, carbamidomethylation of cysteines; variable modifications, oxidation of methionine. Proteins with scores >65 and identified by at least 2 significant sequences, were considered as reliable proteins. Also the corresponding bands in the control sample gel line were analyzed with the same protocol. The proteins detected in treated samples but not in controls were taken into account as possible oridonin binders. This DARTS experiment was performed in triplicate, and the proteins identified in all the experiments were considered putative oridonin targets. A similar procedure was performed to carry out DARTS on living cells. Jurkat cells (2 × 10^6^ cells/well) were treated with 5 μM of oridonin or 0.1% DMSO for 2 h at 37 °C. Cells were lysed with RIPA buffer supplemented with protease and phosphatase inhibitors. After centrifugation and determination of protein concentration, each lysate (50 μg) was diluted with PBS, quickly warmed to R.T. and proteolysed with subtilisin (enzyme: lysate 1:1000 w/w) for 30 min. Reaction mixture was then analyzed as described above. Also in this case, DARTS experiment was performed in triplicate, and the proteins identified in all the experiments were considered putative oridonin targets.

### Chemical proteomics

Oridonin was immobilized on a TentaGel resin to perform chemical proteomics experiments. About 300 μg of HL-S-Trityl (FLUKA-Sigma Aldrich) resin was washed with 100 μL of MeOH/TFA/TIS (94:1:5 v:v:v) for three times, and then activated with 100 μL of 5% TFA in MeOH. The activated resin was incubated with 4 μg oridonin (12 nmol, resin/oridonin molar ratio 1:1) and the mixture was maintained under continuous shaking over night at R.T. HPLC analysis of the reaction solution allowed evaluating an immobilization yield of 70%. Jurkat cell lysates (400 μg) were incubated with the oridonin-modified resin, or with a control resin (HL-S-Trityl incubate overnight with β-mercaptoethanol) for 2 h at 4 °C under shaking. The beads were washed three times with PBS and interacting proteins were eluted by 15 μL of Laemmli buffer 4×. Eluted proteins were separated on a mono-dimensional 10% SDS-PAGE and stained with Brilliant Blue G-Colloidal. Each gel line was cut in 10 pieces, digested and analyzed as describe above. Chemical proteomics experiment was performed in triplicate, and the proteins identified in all the experiments were considered putative oridonin targets.

### SPR analysis of Nucleolin complexes

SPR studies on the interaction between oridonin and Nucleolin were performed using an optical biosensor BIACORE 3000 (GE Healthcare, Milano, Italy). Recombinant human Nucleolin was immobilized on a CM5 sensor chip using a 1 μM protein solution in sodium acetate 50 mM, pH 4.5. using a standard amine-coupling protocol. Oridonin was dissolved in 100% DMSO to obtain 4 mM solutions, and diluted in PBS (10 mM NaH_2_PO_4_, 150 mM NaCl, pH 7.4) to a final DMSO concentration of 0.5%. The binding study was performed using a five-point concentration series (1, 5, 25, 125 and 625 nM and triplicate aliquots of each compound concentration were dispensed into single-use vials. Binding experiments were performed at 25 °C, using a flow rate of 50 μL/min, with 60 s of association and 300 s dissociation monitoring time. Simple interactions were adequately fit to a single-site bimolecular interaction model, yielding a single K_D_. Sensorgram elaboration was performed using the BIAevaluation software provided by GE Healthcare.

### CETSA experiments

Protocol used was adopted from the literature^22^, but it was slightly modified. Approximately 1 × 10^6^ of Jurkat cells in a final volume of 10 mL of growth media were used for each condition, in a Cell Culture Flasks (T75). A stock solution of oridonin (10 μL of a 20 mM in DMSO) was added to individual flasks to get a final oridonin concentration of 5 μM (0.1% DMSO); 10 μL DMSO was used as control. Cells were gently mixed by pipetting up and down at least 3 times and were incubated for 1 h up to 4 h in the CO_2_ incubator at 37 °C. After, the cell suspensions were collected and centrifuged in 15 mL conical tubes. Cell pellets were washed with PBS and gently suspended in 1 mL of PBS supplemented with protease inhibitors. Each cell suspension was divided into 10 different tubes and heated in a PCR machine (Invitrogen Life Science Technologies) at different temperatures (40, 43, 46, 49, 52, 55, 58, 61, 64, 68 °C) for 3 min. After heating, tubes were kept at R.T. for 3 min and then immediately snap-freezed in liquid nitrogen. After that, cell lysate–containing tubes were centrifuged at 20,000 g for 20 min at 4 °C to pellet cell debris together with precipitated and aggregated proteins. Each supernatant (10 µL) underwent western blotting analysis using anti-Nucleolin (rabbit polyclonal, ab22758; Abcam, Cambridge, UK) and anti-GAPDH (rabbit polyclonal, sc-25778; Santa Cruz Biotechnology, Santa Cruz, CA, USA) antibodies. Cell whole lysates for immune-blotting analysis were prepared according to the standard protocol. Protein concentration was determined by DC Protein Assay (Bio-Rad, Berkeley, CA, USA), using BSA as a standard. Proteins were fractionated on SDS-PAGE, transferred into nitrocellulose membranes, and immune-blotted with appropriate primary antibodies. Signals were visualized with appropriate horseradish peroxidase-conjugated secondary antibodies and enhanced chemiluminescence (Amersham Biosciences-GE Healthcare, NY, USA). Densitometry of bands was performed with ImageJ software. The achieved results were plotted on graphs reporting the ratio between the density measured for each Nucleolin band and the density measured for the corresponding GAPDH band. The density measured for cells kept at 40 °C was set as 100%.

To perform the ITDRFCETSA experiments, equal number of Jurkat cells (1 × 10^6^ cells/points) were seeded in 24 well cell culture plates in 1 mL of growth media ad exposed to different concentrations of oridonin (0.25, 0.5, 1, 2.5, 5, 10 and 20 μM) for 2 h. Following the incubation, the drug-containing media were removed by centrifugation; cells were washed with PBS and prepared for CETSA experiment. In this case cells were heated at 55 °C for 3 min, and the experiment was performed as described above. In this case, the density ratio Nucleolin/GAPDH measured at Nucleolin concentration producing the maximum stabilizing effect (10 μM) was set as 100%.

### Cell culture

Jurkat (T-cell leukemia) and HeLa (cervical carcinoma) cell lines were obtained from the American Type Cell Culture (ATCC) (Rockville, MD, USA). Cells were maintained in DMEM (HeLa) or RPMI 1640 (Jurkat), supplemented with 10% FBS, 100 mg/L streptomycin and penicillin 100 IU/mL at 37 °C in a humidified atmosphere of 5% CO_2_. To ensure logarithmic growth, cells were subcultured every two days. All experiments were performed using cells seeded at 2 × 10^5^ cells/mL.

### Immunofluorescence

Control and oridonin-treated Jurkat cells (5 µM FlOr for 2 h) were fixed in a freshly prepared mixture of 4% paraformaldehyde in PBS for 30 minutes and permeabilized with 0.1% Triton X100 for 10 minutes. After washing with PBS, cells were incubated with primary rabbit anti-Nucleolin (1 µg) for 2 h. Following three further washes in PBS, cells were incubated for 1 h with species-specific, fluorescein isothiocyanate (FITC) antibody (Jackson Immunoresearch, UK), at a 1:500 dilution, washed extensively in PBS and finally treated with Hoechst 33342 (10 µg⁄ml) for 15 minutes. Confocal *images* were taken with a Zeiss LSM 510 META confocal microscope with a X63 objective lens.

### Cell viability

The number of viable cells was quantified by MTT ([3-(4,5-dimethylthiazol-2-yl)-2,5-diphenyl tetrazolium bromide]) assay. Absorption at 550 nm was assessed using a microplate reader (LabSystems, Vienna, VA, USA). Cell viability was also checked by Trypan Blue exclusion assay using a Bürker counting chamber.

### Cell cycle

Cell cycle was evaluated by propidium iodide (PI) staining of permeabilized cells according to the available protocol^49^, and flow cytometry (BD FACSCalibur *flow cytometer*, Becton Dickinson, San Jose, CA, USA). Data from 5000 events per sample were collected. The percentages of the elements in G_0_/G_1_, S and G_2_/M phases of the cell cycle were determined using the MODFIT software.

### RNA isolation and quantitative real-time RT-PCR (qRT-PCR)

Total RNA was isolated using Trizol Reagent (Life Technologies, Grand Island, NY, USA) according to the manufacturer’s instructions and spectrophotometrically quantified. RNA integrity was assessed by agarose gel electrophoresis. RNA (3 µg) was reverse transcribed, and real-time PCR was performed with Light-Cycler® 480 (Roche Diagnostics GmbH, Mannheim, Germany) using SYBR Green detection in a total volume of 20 µL with 1 µL of forward and reverse primers (10 mM) and 10 µL of SYBR Green I Master Mix (Life Technologies). Reactions included an initial cycle at 95 °C for 10 min, followed by 40 cycles of denaturation at 95 °C for 10 sec, annealing at 56 °C for 5 sec, extension at 72 °C for 15 sec. The 18S RNA was used as an internal standard. The following primer sets were used for real-time PCR to assay specific mRNAs:

forward Akt 5′-TCT ACA CCC ACA GAT GAC AG-3′

reverse Akt 5′-CTC AAA TGC ACC CGA GAA AT-3′

forward Bcl-2 5′-GGA AGT GAA CAT TTC GGT GAC-3′

reverse Bcl-2 5′-CTC CAT CAG CTT CCA GAC AT-3′

forward 18S 5′-CGA TGC TCT TAG CTG AGT GT-3′

reverse 18S 5′-GGT CCA AGA ATT TCA CCT CT-3′

### Protein synthesis inhibition

Jurkat and HeLa cells were incubated with 5 µM oridonin for 1 h or 2 h and 20 µM oridonin for 2 h,respectively, or with 50 μg/mL cycloheximide (CHX) for 30 minutes, in a cell culture incubator. Cells were then processed for detection of protein synthesis according to the protocol described in the Protein Synthesis Assay Kit (Cayman Chemical, Michigan, USA). Following incubation, the samples were treated with Click-iT® OPP (O-Propargyl-Puromycin), and added of 5-fluoresceina azide, the click reaction reagents leading to a chemoselective ligation between the azide dye and the alkyne OPP, allows the modified proteins to be detected. Finally, all the samples were analyzed by flow cytometry.

### Statistical analysis

Data are reported as the mean values ± SD from at least three experiments, performed in duplicate (n ≥6), showing similar results. Differences between treatment groups were analyzed by Student’s t-test. Differences were considered significant when p < 0.05.

## Electronic supplementary material

Supplementary information

## Data Availability

The datasets generated during and/or analysed during the current study are available from the corresponding author on reasonable request.
