# Supplementary material for: The anti-tumor diterpene oridonin is a direct inhibitor of Nucleolin in cancer cells
Source: Sci Rep. 2018 Nov 13;8:16735. doi: 10.1038/s41598-018-35088-x (PMC6233161; doi:10.1038/s41598-018-35088-x)
Supplement: Supplementary file 1 — Supplementary information [file 41598_2018_35088_MOESM1_ESM.pdf]

The anti-tumor diterpene oridonin is a direct inhibitor of Nucleolin in cancer cells

Michele Vasaturo, Roberta Cotugno, Lorenzo Fiengo, Claudio Vinegoni, Fabrizio Dal Piaz & Nunziatina De Tommasi

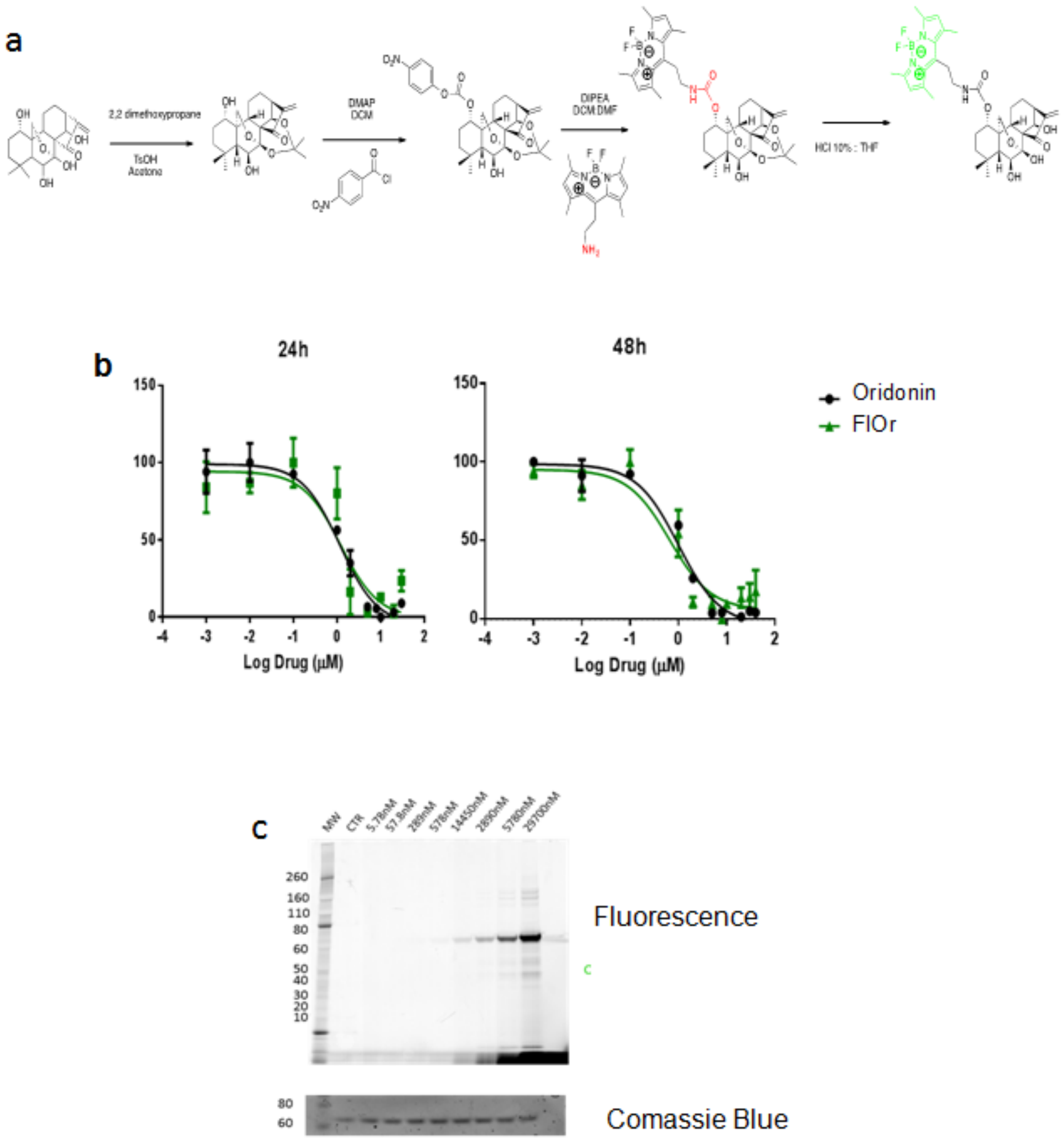

Supplementary figure S1: FlOr synthesis and characterization. Synthethic route for FlOr (a); comparison of tumor cell (Jurkat cell line) growth inhibition by oridonin and FlOr evaluated following 24 h and 48 h of incubation (b); Denaturing gel electrophoresis of increasing concentration of FlOr incubated with purified HSP70 for 15 min and imaged with a fluorescence gel scanner using 488 nm excitation/512 nm emission (c).

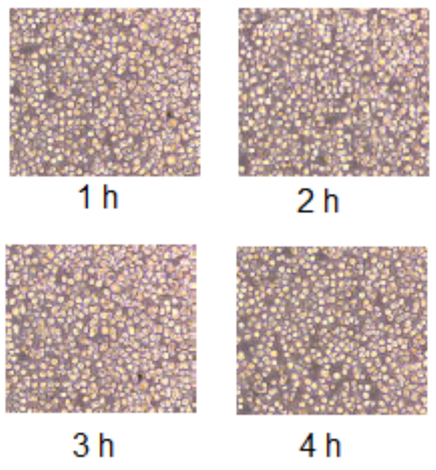

Supplementary figure S2: **Light pictures of Jurkat cells incubated with FlOr for different times**. The same cells were analysed by fluorescence microscopy (Figure 2).

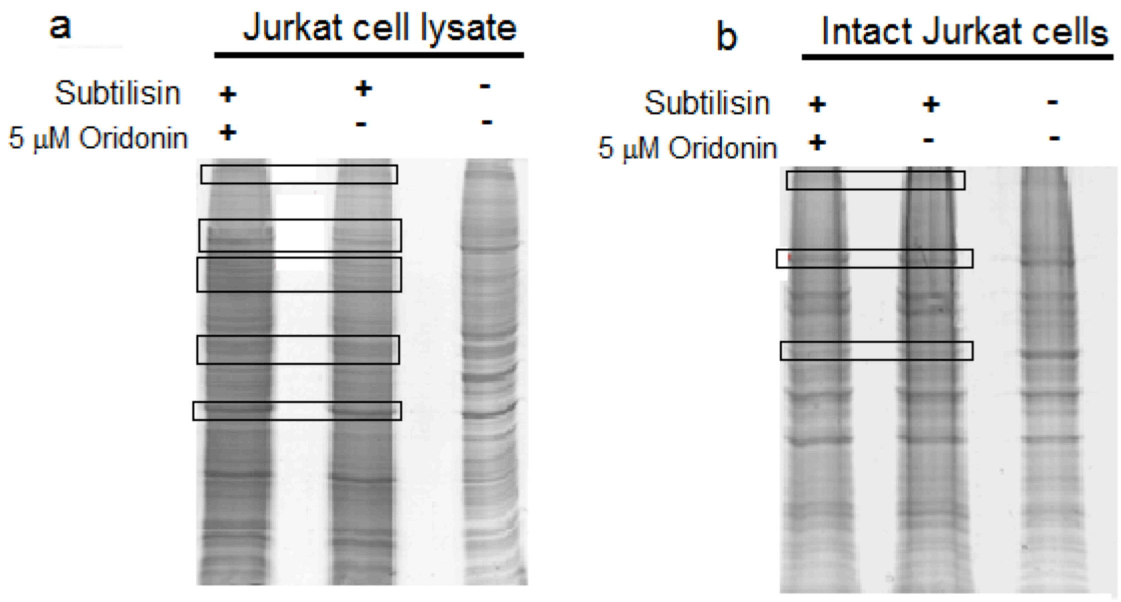

Supplementary figure S3. SDS-PAGE separation of protein from Jurkat cells following DARTS experiments. Experiment on cell lysate (a): lane 1) cell lysate treated with oridonin 5 µM for 1h and then digested; lane 2) cell lysate treated with DMSO for 1h and then digested; lane 3) Jurkat cells undigested lysate. Experiment on intact cells (b): lane 1) cells treated with oridonin 5 µM for 2h and then digested; lane 2) cells treated with DMSO for 2h and then digested; lane 3) Jurkat cells undigested lysate. The gel bands subjected to trypsin digestion were boxed in black.

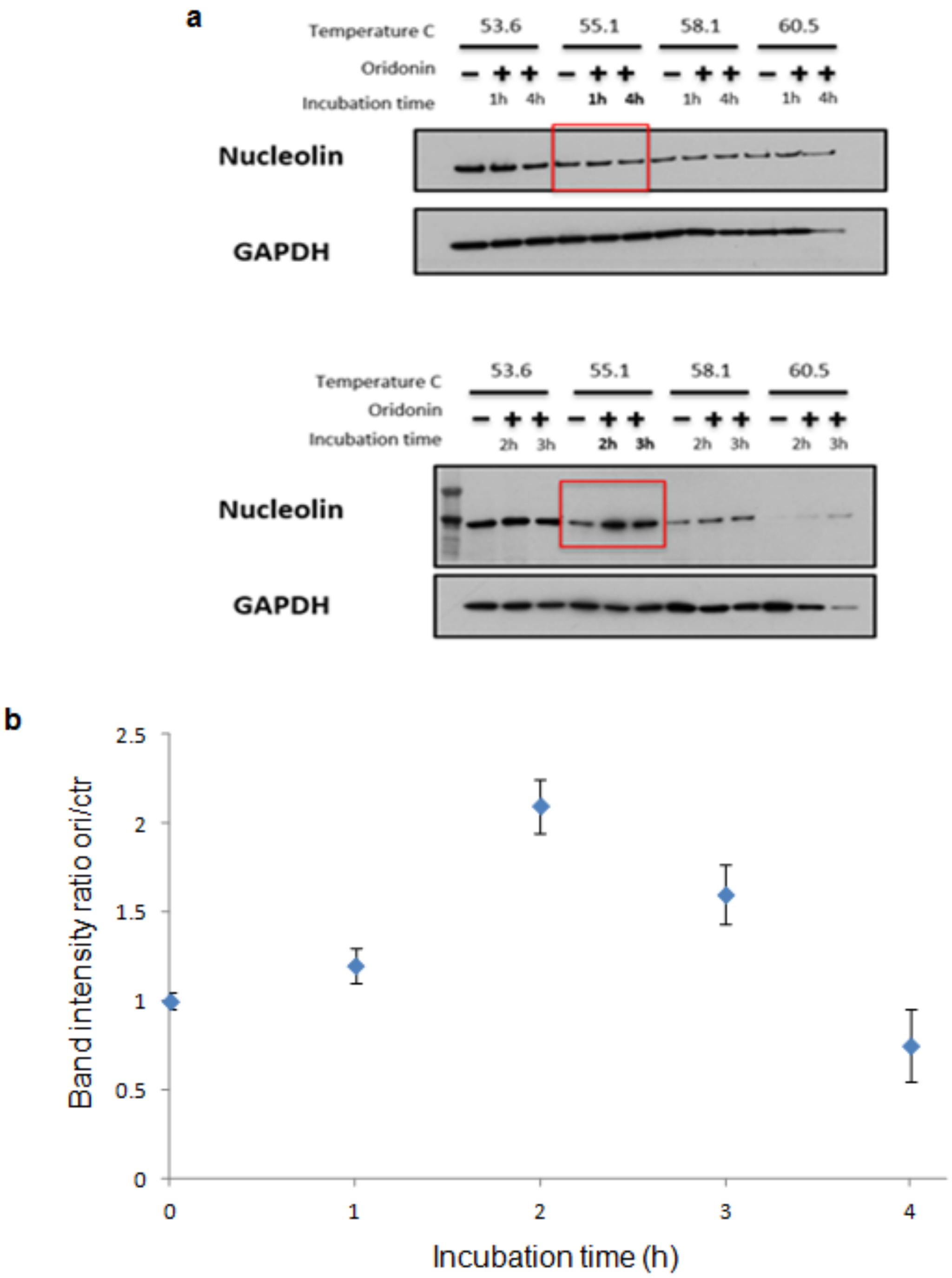

Supplementary figure S4. **Kinetic profile of the engagement of Nucleolin by oridonin in cells using CETSA.** Jurkat cells were treated with oridonin 20 µM for 1h up to 4h. (a) Each samples was heated and then the soluble amount of NCL was evaluated by western blotting analysis. (b) Soluble Ncl amounts observed at 55 °C following different incubation times were reported in the graph. Blots of Nucleolin, and GAPDH were taken from different gels.

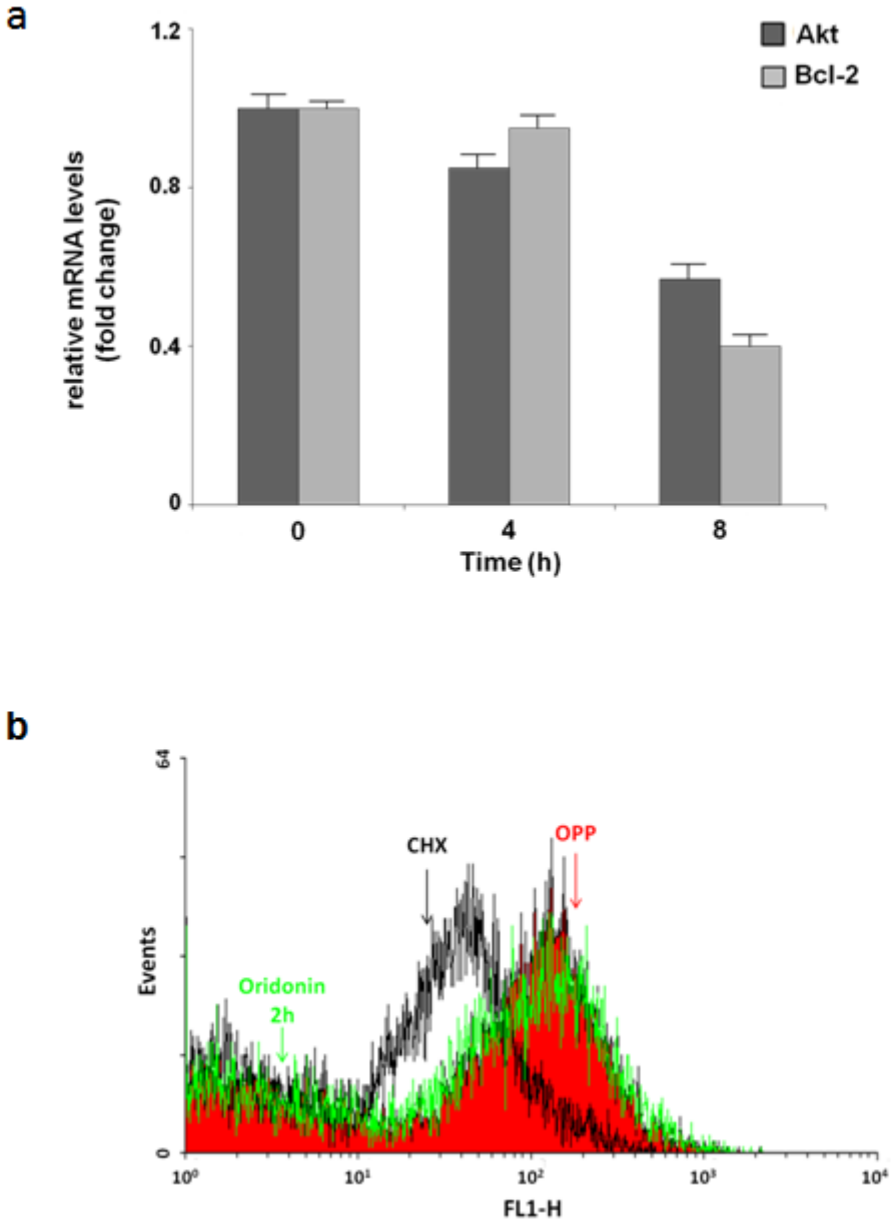

Supplementary figure S5. Oridonin effect on Ncl mRNA and protein synthesis. (a) Akt and Bcl-2 mRNA levels in HeLa cells treated with 20 μM oridonin for 4 and 8 h. Data are the mean values ± SD from two independent experiments performed in duplicate (n=6). (b) HeLa cells were treated with vehicle (red), 50 μg/ml cycloheximide for 30 min (black) and 20 μM oridonin for 2 h (green). Cells were then incubated with OPP.

Supplementary Table S1: Putative oridonin-interacting proteins emerged from DARTS experiments on Jurkat cell lysate

Swiss-Prot CODE	Score*	Protein	Peptides**
HSP90B_HUMAN	2633	Heat shok protein Hsp90-beta	36
HSP71A/HSP71B_HUMAN	2005	Heat shock 70 kDa protein 1A/1B	21
NUCL_HUMAN	1977	Nucleolin	18
XRCC6_HUMAN	664	X-ray repair cross-completing protein 6	17
CALX_HUMAN	358	Calnexin	16
IMB1_HUMAN	338	Importin subunit beta-1	10
TLN_HUMNA	297	Talin-1	17
ENPL_HUMAN	197	Endoplasim	12
SF3B3_HUMAN	175	Splacing factor 3B subunit 3	11
ILF3_HUMAN	145	Interleukin enhancer-binding factor 3	11
GRP75_HUMAN	144	Stress-70 protein	6
NUP93_HUMAN	93	Nuclear pore complex protein 93	5

* Average score value achieved in three different chemical-proteomics experiments
** Number of unique peptide sequences detected in a single experiment. Reported values are the average of the unique sequences detected in three different chemical-proteomics experiments

Supplementary Table S2: Putative oridonin-interacting proteins emerged from DARTS experiments on intact Jurkat cells

Swiss-Prot CODE	Score*	Protein	Peptides**
HSP71A/HSP71B_HUMAN	560	Heat shock 70 kDa protein 1A/1B	11
NUCL_HUMAN	459	Nucleolin	9
TCPG_HUMAN	350	T-complex protein 1 subunit gamma	13
HSP90B_HUMAN	201	Heat shock protein Hsp90-beta	6
HNRPK_HUMNA	142	Heterogeneous nuclear ribonucleoprotein K	4
TCPA_HUMAN	72	T-complex protein 1 subunit alpha	4
CH60_HUMAN	58	60 kDa heat shock protein	2

* Average score value achieved in three different chemical-proteomics experiments
** Number of unique peptide sequences detected in a single experiment. Reported values are the average of the unique sequences detected in three different chemical-proteomics experiments

Supplementary Table S3: Mass spectrometry-based identification of peptides generated by trypsin digestion of Nucleolin/oridonin complex

Peptide	Experimental molecular weight	Theoretical molecular weight
10-15	657.316	657.308
17-22	639.353	639.341
23-51	3312.338	3312.299
56-62	618.350	618.334
64-70	728.461	728.443
72-79	755.508	755.454
81-87	642.381	642.370
89-95	628.368	628.354
97-102	615.371	615.359
103-109	672.394	672.381
117-124	755.469	755.454
126-132	626.402	626.375
143-176	3990.583	3990.341
177-217	4445.596	4445.553
231-274	5277.803	5277.712
289-294	585.320	585.312
298-318	2311.174	2311.148
319-324	643.377	643.354
325-333	936.511	936.492
334-342	999.602	999.535
343-347	576.331	576.305
349-362	1647.799	1647.730
363-370	843.575	843.506
371-377	805.491	805.433
378-382	613.402	613.380
399-403	633.387	633.349
404-410	831.497	831.434
411-420	1177.603	1177.561
430-437	939.551	939.506
438-444	762.379	762.339
450-457	874.437	874.414
458-467	1159.611	1159.576
468-474	879.406	879.383
478-486	994.495	994.436
487-508	2500.303	2500.258
514-521	883.479	883.451
524-537	1593.788	1593.735
538-545	877.416	877.396
555-561	811.508	811.455
562-567	600.320	600.298
573-577	606.416	606.374
578-589	1321.691	1321.625
590-597	895.444	895.403

605-610	607.312	607.218
611-624	1560.738	1560.673
628-639	1306.593	1306.535
640-648	1056.627	1056.597
649-656	735.366	735.330
695-705	1036.541	1036.505
